# Analysis of Digital Competence for Spanish Teachers at Pre-University Educational Key Stages during COVID-19

**DOI:** 10.3390/ijerph18158093

**Published:** 2021-07-30

**Authors:** Esteban Pérez-Calderón, Jorge-Manuel Prieto-Ballester, Vanessa Miguel-Barrado

**Affiliations:** 1 Faculty of Economics, University of Extremadura, 06006 Badajoz, Spain; 2 Faculty of Education, University of La Rioja, 26006 Logroño, Spain; jorgemanuel.prieto@unir.net; 3 Faculty of Law, University of Extremadura, 10004 Cáceres, Spain; vmiguel@unex.es

**Keywords:** pre-university education, COVID-19, digital competence, ICTs

## Abstract

Over time, the role of information and communication technologies (ICT) has become increasingly important in most areas of our lives, including education. So much so, that during the COVID-19 pandemic, the use of these tools has been essential to continue the teaching process. One of the great challenges facing teachers today is their need to adapt to this new educational scenario by acquiring the necessary digital skills. The aim of this study is to determine the level of competence of teaching in pre-university education key stages. To this end, a questionnaire was distributed among education centres and teachers in the Autonomous Community of Extremadura, obtaining 109 valid responses. The analysis methodology was the formation of clusters using the K-means model. The results confirmed that the teachers perceived a medium-high level of knowledge and use of ICT. Moreover, that this digital competence is conditioned by factors such as age, experience, gender, and level of education. In conclusion, public administrations are encouraged to facilitate teachers’ knowledge and application of ICT according to the profiles identified.

## 1. Introduction

The use of resources based on information and communication technologies (ICT) has changed the way we relate to each other [1], leading to an increase in communication between family and friends [2]. In the labour market, knowledge and competence in the use of ICT are key elements in any type of activity sector. However, only 26% of organisations are prepared to face the changes brought about by new technologies [3].

These advances in technology have also been slowly transferred to the education system [4], providing teachers with both material and immaterial resources, beyond methodological improvements, which have allowed them to deepen the teaching/learning process, facilitating this work [5,6].

Competence in the use of ICT has been considered a basic skill required of students and so it should also be a fundamental requirement for teachers [7]. Furthermore, teachers are faced with a typology of students who are joining the education system with an increasingly higher skill level of ICT use [8]. Thus, a problem may arise if the people who must lead the teaching/learning process have not experienced the development of ICT at the same level of intensity as their students. Today’s teachers cannot be classified as digital natives, but their ICT skills are based on the knowledge that they have had to acquire throughout their working and personal lives.

Thus, many doubts may arise as to whether the level of competence achieved is sufficient for teachers to be able to lead a process in which the real protagonists of their work may have a higher level of skill. In some cases, we even find that, with an adequate level of ICT training, the transition to the implementation of these processes in the teaching environment exposes many gaps within the teaching staff themselves and a lack of confidence in their use [9,10,11,12].

Additionally, although the use of ICT resources has been considered an important element in the development of teaching for a number of years, recent global events have shown that the use of ICT should be a fundamental requirement for dealing with exceptional situations such as the one being experienced with COVID-19, and which may anticipate in time a change in the paradigm for the educational model conceived to date [13]. This pandemic has meant that teaching has gone from being mostly face-to-face to being virtualised at a rapid pace to all levels of the educational community [14,15]. This obligation to virtualise teaching has revealed many shortcomings that might have been thought to have been overcome and which will surely soon be addressed by the academic world.

It has already become clear that digital competence cannot be seen only as an element that allows access to the use of ICT, but also as a key element of participation today [16,17]. Therefore, the justification for studies on the role of ICT in the classroom, which was already sufficient before, has been reinforced with the new crisis caused by the global pandemic, where the virtualisation of teaching has gone from being an option to being the only option.

Internationally, there has been a deep concern that training has moved from the mere transmission of knowledge to the acquisition of skills by students. This concern has been led by institutions themselves [18,19,20,21,22,23]. There are several models of reference for teachers’ digital competences. One of them is the TPACK model. This model integrates technical knowledge, pedagogical knowledge, and the use of ICT to shape meaningful learning [24]. In order to achieve this meaningful learning, it is essential that teachers have sufficient digital competences [25,26]. This model enables the appropriate use of technology by teachers, as well as their professional development [26].

Among other models proposed by institutions, those designed by the European Commission and UNESCO can be highlighted [24]. Undoubtedly, the international commitment to the integration of ICT in education has been manifested in different programmes such as DigCompEdu or UNESCO’s ICT competences framework [14]. Both models detail ICT competences in teachers, from a European and global perspective, respectively [24]. Thus, the DigCompEdu programme seeks the integration of ICT at all levels of education by addressing pedagogical, technological, and organisational spheres. In order for its implementation to be effective, the different particularities of each educational sector, such as autonomy, research, or innovation, must be taken into account [14]. The framework proposed by UNESCO is a primarily organisational model, focusing on didactic issues and the incorporation of technology into curricula, teacher training, and educational organisation [24]. Furthermore, the development of both frameworks is fully compatible and can complement each other [14].

The current literature highlights the benefits of skills-based learning [19], which has led to it being an extremely important element in curriculum development in Spain in recent years [27], with digital competence being considered one of the basic skills of the education system [6,17]. In the previous approach, it is also necessary to consider that technologies are constantly changing, making them obsolete in a very short time. This causes a situation of disorientation for teachers, leading to certain insecurities when it comes to development in ICT environments [28].

ICT is an element that is yet to be exploited at the same level in educational settings as in other social and essential areas, and therefore its scope and growth are still quite broad. There is still a long way to go to achieve adequate initial training for teachers, as well as the professional development that this may entail for them [24].

The use of ICT is not only justified by the intrinsic need that the pandemic has provoked in the education system but also because multiple advantages justify it, such as [29,30,31,32]:

- Improves knowledge acquisition.
- Acts as a motivating element.
- Favours autonomous learning.
- Enables the expansion of knowledge.
- Facilitates participatory methodologies and the development of distant or asynchronous teaching.

The mere fact of the existence of ICT does not change the educational process, instead, it necessary to pay attention to the level, and above all the manner, of its implementation and teacher training for its proper use [24,29]. In order to establish this strategy for the improvement and acquisition of digital skills by teachers, it is essential to know their initial level of these skills [17,33,34,35,36,37,38].

For all the above reasons, it is important to define a model so that teachers can acquire adequate competence and overcome the gap between their knowledge and the actual implementation of ICT in their teaching methodology [10,39,40,41,42]. This study aims to assess the level of teacher digital competence (TDC) in pre-university Spanish education key stages (*Educación Secundaria Obligatoria*, ESO, equivalent stage to UK years 8–11; *Bachillerato*, B, and *Formación Profesional*, FP, equivalent stages to UK years 12–13) using the questionnaire by Tourón et al. [37], validated and based on the competencies established by the NIETTT (National Institute of Educational Technologies and Teacher Training) in the European Union’s Common Digital Teaching Framework, to find out how teachers perceive themselves concerning TDC.

In the review of the literature on the subject, it has been found that most studies do not specify the level of TDC of teaching staff at pre-university key stages, which makes studies of this type even more necessary. Therefore, to expand the evidence on the relationship between the knowledge and use of ICT in teaching staff at pre-university educational key stages, one main objective and three specific objectives have been set. The main objective is to ascertain the level of teaching digital competence achieved by pre-university education teaching staff. Parallel to this main objective, we can find other secondary objectives such as: classifying teachers according to their digital competence; identifying training gaps related to the TDC; and proposing educational policies aimed at improving the TDC.

Part of the interest of this work lies in the importance of generating prior knowledge to establish training policies, i.e., to serve as a reference for the design of teacher training policies aimed at identifying the strengths and weaknesses of teachers and redirecting future training in everything related to the improvement of the TDC.

## 2. Materials and Methods

This study is framed within the model established by the European Union (DigComEdu) being applied in Spain through the NIETTT enhancing the pedagogical sphere [24]. This institution has tried to respond to the level of ICT qualifications needed by Spanish teachers in the European reference framework [43].

The present study is quantitative, within a non-experimental design that has not modified any of the study variables initially proposed. It, therefore, aims to achieve the proposed objectives based on the questionnaire validated by Tourón et al. [37]. The initial form is made up of 54 items, grouped into five skill areas, which allow us to find out the vision that teachers at pre-university educational key stages have of their knowledge and application of ICT. The sample consisted of teachers in the Autonomous Community of Extremadura. Convenience sampling was used and participation in the questionnaire was voluntary. A total of 109 valid responses were obtained, of which 43 (39.45%) were men and 66 (60.55%) were women. Table 1 shows the representation by age group, gender, and teaching experience (see Table 1).

The following tables show the descriptive statistics for the variables used in the study (see Table 2 and Table 3).

The choice of the questionnaire has been highlighted within the theoretical framework. Without wishing to repeat what has already been justified, it should be noted that this questionnaire has already been validated by a group of experts and tested with 426 teachers [37]. The questions have been grouped according to the area to which they refer to make the responses much simpler; these groupings coincide with the areas of assessment of the TDC.

The variables used to define the teachers’ profiles are listed in the Table 4.

Recent studies in the literature have used some of the variables used in this study such as gender, years of experience, and key stage [44,45].

The procedure for collecting responses was carried out through various channels. On the one hand, the questionnaires were sent to high schools so that they could disseminate them among their teaching staff. On the other hand, the questionnaires were disseminated through social networks such as Facebook and Twitter. In addition, the questionnaire was sent to active teachers, with whom we had contact so that they could disseminate it among their acquaintances and colleagues. This phase of questionnaire distribution took place between 20 and 30 May 2020. The questionnaire has been implemented using the Google Forms tool, which allows us to monitor the responses received in Excel format in real-time, to facilitate their download and processing.

The methodology applied in this study was a K-means group analysis. Previously, a descriptive analysis and Spearman’s correlation analysis were performed.

The data analysis was carried out with the outputs obtained with the SPSS statistical package (v.22).

## 3. Results

In a first approach, for the study of the TDC, a purely descriptive analysis was carried out for each category of items. Beginning with the set of items representing the skills associated with the problem-solving variable (see Table 5), the teachers displayed the highest scores regarding management and storage in the cloud (PST01) and tools for carrying out assessments, tutorials, or monitoring students (PST05), both in terms of knowledge and application of these skills (5.84 and 5.71; 5.14 and 4.94, respectively). The lowest mean score, both in terms of knowledge and use of the skill, was item PST12, corresponding to the forms of peer-to-peer problem solving (3.76 and 3.27, respectively).

For the set of items associated with tools for accessing and managing information (see Table 6), teachers showed greater knowledge and greater use of strategies for navigating the Internet (INF01: 5.40 and 5.18, respectively). The worst skill, both in terms of knowledge and use, was the item referring to tools for recovering deleted or damaged files or those affected by any formatting problem (INF06: 3.36 and 3.05, respectively).

Of the numerous indicators that make up the variable of teaching competencies for content creation (see Table 7), the high scores for knowledge and use of tools for creating presentations (CCT04: 5.60 and 5.21, respectively) stand out. On the contrary, the low evaluation obtained in the question referring to knowledge and use of tools for content based on augmented reality (CCT05: 2.83 and 2.01, respectively) deserves special mention.

In the variable representing competence in communication tools, most of the responses reported a medium-high rating. Respondents expressed a high valuation in the knowledge and use of online communication tools, such as forums, video calls, or instant messaging (see Table 8, COM03: 5.80 and 5.56, respectively). In the second place, in terms of knowledge and use, were tools associated with the communication of grades and the evaluation of assignments or tutorials (COM02: 5.69 and 5.50, respectively). In this category, the high level of knowledge developed by teachers concerning the use of social networks such as Facebook or Twitter (COM05) stands out. Finally, the lowest knowledge scores were achieved for the tools regarding the management of digital identities in the educational context (COM06).

In the category referring to tools related to safety and the proper use of technology, Table 9 shows how the highest level of knowledge and use referred to systems for accessing and protecting devices or documents (SAF01: 5.02 and 4.85, respectively). The rest of the items reported a medium-high valuation, with the aspect referring to knowledge and use of means of control between technology and student distraction obtaining the lowest score (SAF08: 3.58 and 3.23, respectively).

As an extension of the descriptive analysis, the results obtained in the Spearman correlation analysis showed a positive and highly significant association for most of the items between the variables age and experience (see Appendix B, Table A1, Table A2, Table A3, Table A4, Table A5, Table A6, Table A7, Table A8, Table A9, Table A10, Table A11, Table A12, Table A13, Table A14, Table A15 and Table A16). In particular, for the categories of ICT tools including problem solving, content creation, information access and management, and communication. The female gender is also associated with lower levels of some digital skills, especially in the case of problem solving (see Table A7 and Table A12), information access and management (more in knowledge than in use, see Table A8), safety (more in knowledge than in use, see Table A11), and communication (more in knowledge than in use, see Table A11).

In addition to the previous descriptive study, a K-means cluster analysis was performed to classify the data according to the variances observed in the responses. Thanks to this method, homogeneous groups were obtained in which to observe characteristics and define different teacher profiles according to their knowledge and use of ICT skills. The method also ensures the maximum difference between the variances between the groups formed. In our case, checking the result of the ANOVA analysis, all the items are significant (*p* value < 0.01) in their contribution to the definition of the group in which they are classified (see Appendix C, Table A12, Table A13, Table A14, Table A15 and Table A16). This is because we have worked with a refined and contrasted model in previous studies [37]. When setting the number of groupings, the dendrogram was used as a reference once the responses were classified in the different items for each competence category and according to Ward’s method.

Beginning with the category referring to knowledge of communication tools (K_COM), Table 10 shows that two well-differentiated groups were obtained. In one of them, almost 75% of the total are classified as showing a high use of these tools (5.56 out of 7). The rest show a clear deficiency with an average rating of 3 out of 7. In the first case, these are teachers with an average age and experience of 43.63 and 12.43 years, respectively. Age seems to be a determining factor in the knowledge of these tools, with those over 50.64 years of age being the least knowledgeable. Women show a higher level of knowledge, representing 54.3% within the highest scoring group. By type of school, most of the teachers with the highest level of knowledge work in public schools and urban environments (87.7% and 60.5%, respectively). Additionally, higher educational stages (sixth form 7 A’ Level) bring together a greater number of teachers with greater knowledge of these tools (66.7%).

According to the teachers’ declared use of ICT communication tools, three groups (U-COM) were obtained. As can be seen in Table 10, the level of use is lower than the declared level of knowledge. In one of these groups are teachers who have declared a lower level of use of these tools, 35% of those surveyed, obtaining an average score of 3 out of a maximum of 7. The profile of the teachers who reported a lower use of these types of tools corresponds to teachers of older age and average experience; they are teachers who teach at the highest educational level, i.e., years 12–13, and the proportion of women is higher (66.7%). The rest of the teachers achieve a notable rating. In one of the two groups with the highest levels of knowledge, there are deficiencies related to the management of digital identities, the sharing of experiences or educational research of other colleagues, and tools for collaborative learning (see Table 10).

Analysing the content creation group, in terms of teachers’ knowledge (K-CCT), two well-differentiated groups were obtained (see Table 11). In one of the groups, with a representation of 36.7%, the teachers failed (2.56). This group has a higher average age, while the other group has a notable level of knowledge (5.31). Once again, age conditions ICT knowledge. The group with the lowest scores is made up mostly of women, teachers in urban and public schools, and at higher educational stages, as opposed to those who teach ESO (years 8–11). Within the group of teachers who best value their knowledge, a more uniform number of cases can be observed according to gender, school type (rural/urban), or educational stages. However, the province of Badajoz and public schools are those with the highest number of teachers in this group.

A similar result was obtained in the category referring to the use of this type of tool (U-CCT, see Table 11), with younger teachers reporting greater use compared to teachers with a higher average age, even though these teachers have more experience. Although three groups were obtained, one of them can be discarded since it was formed by a single teacher who can be considered an atypical case for the rest. The group with the highest number of teachers is the one that fails in the use of this type of ICT (58.72%), with women, teachers in public and urban schools, and at higher educational stages (years 13–14) highlighted. In the group with the highest scores, the public ownership of the schools (86.4%) and higher key stages (59%) stand out.

Analysing the characteristics of the groups trained in the knowledge of tools for information access and management (K-INF, see Table 12), the data referring to age and years of experience are significant, with teachers with a lower average age and fewer years of experience (42.86 and 11.42 years, respectively) having the greatest knowledge of information access and management tools. The two groups with the highest reported knowledge account for more than 80% of the respondents. Conversely, those with older age and with more years of experience on average (50.35 and 19.8, respectively) report poor knowledge. Among the teachers with the least knowledge of these information resources, it is noteworthy that the vast majority are women (80%). In addition, the data show that teachers who teach in public schools, in urban areas, and in the Baccalaureate and Vocational Training field are those who have least valued their knowledge in this area (85%, 70%, and 85%, respectively).

In the category referring to the use of these tools, the results show a significant difference in terms of the average age and experience of the respondents. Younger teachers and those with fewer years of experience (43.44 and 12.78, respectively) use these resources more. Older and more experienced teachers (47.51 and 16.84, respectively) reported less use. Additionally, within this group of teachers, the high percentage of women (73%) stands out. In addition, the results contrast with the fact that the educational level at which these tools have been used the least was in public schools and BFP, years 12–13 (83.8% and 70.2%, respectively).

In the table showing the results referring to knowledge in safety issues (see Table 13), the youngest teachers and those with the least years of experience (43.57 and 12.39, respectively) are the ones with the highest knowledge in this area. This group with the highest rating accounts for 62.38%, while those who are older and have been working for more years are, on average, rated their knowledge of these skills the lowest (48.51 and 18.48, respectively). Another relevant aspect is the high percentage of women among the teachers with the least knowledge of safety (73.2%); as are teachers in public and urban schools and at BFP (85.4%, 68.3%, and 75.6%, respectively). On the other hand, most of the teachers with the most knowledge, 62.38%, teach at the highest levels and in public and urban schools (85.7% and 64.3%, respectively).

In the cluster referring to the use of safety tools (see Table 13), it can be observed that the average age and years of practice differ quite a bit, with the age and years of experience being lower in the most qualified teachers (43.45 and 12.25, respectively) for the teachers who have made the least use of them (48.6 and 18.57, respectively). In addition, there is an important difference within the teachers who have used these tools the least in terms of gender, with the percentage of women being considerably higher (71.4%), mostly in public, urban, and BFP (83.3%, 66.7%, and 73.8%, respectively).

The teachers with the greatest knowledge of problem solving (K-PST, see Table 14) stand out for their younger age and years of average experience (43.23 and 12.02, respectively), while those with the least knowledge in this regard are older and have more years of professional experience (50.72 and 21.09, respectively). It should be noted that the group with the best assessment is the largest, accounting for 70.64% of the total. It is also important to note that most of the teachers with the least knowledge are women (75%), as well as teachers in public, urban, and last educational years (87.5%, 71.9%, and 78.2%, respectively). In addition, the data show that the highest percentage of teachers with high knowledge coincides with the profile of the group with the lowest qualification level.

Again, looking at the statements of use of ICT and problem solving skills, Table 14 shows that teachers with lower average age and professional trajectory are those who have made more use of these problem solving tools (43.69 and 12.33, respectively), to the detriment of those who are older and more experienced (48.56 and 18.92, respectively). The largest group, 70.64%, is formed by those with the best knowledge. Again, within the group with greater deficiencies, women, teachers in public and urban schools, and at higher key stages make up the majority (74.4%, 84.6%, 69.2%, and 71.8%, respectively).

## 4. Conclusions

The study analyses the level of TDC in pre-university teachers (secondary school, years 8–13). Evaluations of indicators associated with the use and knowledge of five dimensions of ICT have been analysed: problem-solving, access and management of information, content creation, communication tools, and safety.

According to the results of the analysis, teachers at these educational levels are not only familiar, but also use tools to make presentations of their content. In addition, tools for cloud management, Internet browsing, synchronous/asynchronous virtual communication, or protection of devices and documents are the skills with which teachers are valued to a greater extent, both in their knowledge and in their use. On the other hand, for peer problem solving, recovery of deleted material, technology control, and student distraction, the lowest ratings were observed and, therefore, there is greater room for improvement, both in terms of knowledge and use. A deficit was detected for content creation tools based on augmented reality, since it is a novel resource and requires a high level of training to generate the necessary skills, this state of affairs having been highlighted before in previous work [42]. It is surprising that teachers express a high level of use of social networks and yet have no knowledge of how to use them in their profession. Undoubtedly, action is needed in this area since they are communication channels used extensively by students in their social activities and their use in education can be a motivation because they are tools that they already master.

Likewise, it has been observed that ICT knowledge and use are conditioned by factors such as age, gender, years of experience, or educational stage. In line with previous studies, digital competence is higher in younger teachers [46,47]. In line with other studies [48,49], a significant difference in terms of gender has been observed within the groups of teachers, with women generally being less skilled in digital competencies and how to use them. Furthermore, according to recent studies [50], the results obtained show that the educational level at which teaching is provided is a factor that has an impact on the level of digital competence of the teachers.

We can conclude that teachers consider that they have an intermediate–high level of knowledge and use of the tools that define their TDC. This good level of knowledge and use has had an impact on their rapid adaptation to the demands of the new educational environment in the worst moments of the SARS-Cov-2 pandemic. In line with the above, the high level of ICT use associated with leisure, relationships, and personal communication has facilitated the use of those ICTs used in the development of their profession as teachers, although further training in the use of these tools in these educational stages is still pending.

It is essential to objectively evaluate the extent to which ICTs have made inroads in education. Therefore, in addition to evaluating the knowledge and use of ICT by teachers, it would be interesting to assess whether this application is carried out from a constructivist point of view and not as a mere instrument within the teaching-learning process.

In addition, one of the key elements that must be considered in the study of teachers’ digital competence is motivation. As in other areas of teacher training, historically, teachers have not had a real motivation for continuous training [24,51]. There are major shortcomings in the motivation for teacher training, and in the field of ICT, generating teacher burnout in the transition from face-to-face to virtual teaching [52]. The COVID-19 pandemic, in which teachers have had to adapt in a short time, and with great effort, to ICT tools for didactic use [53], has led to greater teacher burnout [52].

Other studies analyse contextual facilitators for learning activities using ICT [54]. These perspectives are very interesting to consider in any study of teachers’ digital competences. This would undoubtedly be another limitation of our study and future work would need to consider the complex dynamics of the factors that condition the success of digital teaching. Similarly, it would be interesting to include other factors associated with the digital resources of centres, the day-to-day technical support that teachers have at their workplace, the digital equipment of students, and other institutional, organisational, and administrative factors [54].

In the same vein, public administrations must be motivated to a greater extent to carry out a real development in the use and application of ICTs by teachers. To this end, it is essential that, in addition to the technical means, teachers are provided with training and sufficient time to practice, choose, and develop the most appropriate content for their subject and educational level. Thus, we propose the development of comprehensive, collaborative training plans, open over time, allowing the progressive process of acquisition of digital skills adapted to the specific needs of teachers, schools, and students. It is essential to have teaching staff trained in this way so that they can convey this knowledge to students, making them competent in this area and thus enabling them to make proper use of technology, something of an extreme necessity nowadays.

Finally, the findings from this study indicate that this subject requires further research. Although this study has been carried out on teachers in Extremadura, the findings may give insight into the situation at the Spanish level, as teacher training policies tend to be very similar between the different Autonomous Communities, especially in the field of ICT, where there is a body such as the NIETTT in charge of ICT integration in the educational stages prior to university. This institute is also in charge of ICT training, which means that all teachers, regardless of their place of residence, have the same possibilities to acquire digital competence. As this study has only been carried out on a limited sample, it cannot be regarded as representative of teachers either within the entire Autonomous Community of Extremadura or on a national level, thus, the findings obtained cannot be extrapolated and future studies should be conducted on larger samples.

In addition, another possible improvement could be to study other educational levels, such as infant and primary education, to develop preventive measures in ICT training so that teachers could anticipate students’ ICT skills before they reach the pre-university educational key stages. Finally, the number of items ought to be increased, to include a variable referring to the satisfaction or global self-perception that would allow us to elaborate a more complex analysis of the data using the structural equation technique.

## Figures and Tables

**Table 1:** Age, gender, and teaching experience of the study participants.

Age	Gender	Frequency	%	Teaching Experience	Gender	Frequency	%
Under 25 years old	Man	1	0.91%	Less than 5 years	Man	11	10.00%
	Woman	0	0.00%		Woman	11	10.00%
25 to 35	Man	4	3.64%	Between 5 and 15	Man	15	13.64%
	Woman	2	1.82%		Woman	28	25.45%
35 to 45	Man	20	18.18%	Between 15 and 25	Man	10	9.09%
	Woman	33	30.00%		Woman	11	10.00%
45 to 55	Man	12	10.91%	Between 25 y 35	Man	5	4.59%
	Woman	20	18.18%		Woman	11	10.00%
Over 55 years old	Man	6	5.50%	More than 35 years	Man	2	1.82%
	Woman	11	10.00%		Woman	5	4.55%

Source: Authors.

**Table 2:** Scale variables.

	N	Range	Min	Max	Mean	St. Desv.	Asym.	Kurt.
AG	109	36.0	31.0	67.0	45.57	8.0489	0.478	−0.442
EX	109	50.0	1.0	51.0	14.69	10.9313	0.846	0.006

Source: Authors.

**Table 3:** Nominal variables.

	Group	Freq.	%	Asym.	Kurt.
SX	Man	44	40.4	−0.398	−1.876
	Woman	65	59.6		
PR	Badajoz	83	76.1	1.244	−0.461
	Cáceres	29	23.9		
TC	PF	14	12.8%	−2.252	3.129
	P	95	87.2%		
LC	R	39	35.8%	−0.602	−1.669
	U	70	64.2%		
KS	ESO	33	30.3%	−0.070	−1.577
	BFP	76	69.7%		

Source: Authors.

**Table 4:** Variables.

Age (*AG*): teacher’s age
Gender (*SX*): male or female, with the option of not answering
Experience (*EX*): years of teaching experience
Province (*PR*): Badajoz or Cáceres
Type of Centre (*TC*): public, P; private or funded, PF
Location (*LC*): the school is in a rural or urban area
Key Stage (*KS*): ESO, key stage 3–4, years 8–11; BFP, sixth form 7 A’ Level

Source: Authors.

**Table 5:** Problem solving tools (PST).

	Knowledge		Usage	
Item	Mean	St. Desv.	Mean	St. Desv.
PST01 (cloud storage)	5.84	1.467	5.71	1.635
PST02 (use of digital devices)	4.80	1.983	4.55	2.006
PST03 (diversity in classroom)	4.07	1.849	3.63	1.829
PST04 (digital competence training)	4.99	1.754	4.44	1.802
PST05 (evaluation, tutoring, or monitoring)	5.14	1.795	4.94	1.761
PST06 (computer maintenance)	4.72	2.074	4.46	2.17
PST07 (incorporation of new devices)	4.91	1.788	4.42	1.856
PST08 (student digital competency)	4.73	1.804	4.43	1.791
PST09 (peripheral compatibility)	4.90	2.009	4.74	2.048
PST10 (digital and non-digital technology)	4.88	1.883	4.77	1.916
PST11 (educational project adaptation)	4.44	1.880	4.15	1.873
PST12 (peer-to-peer problems)	3.76	1.865	3.27	1.934

Source: Authors.

**Table 6:** Information access and management tools (INF).

	Knowledge		Usage	
Item	Mean	St. Desv.	Mean	St. Desv.
INF01 (internet browsing)	5.40	1.617	5.18	1.634
INF02 (information management)	4.49	2.010	4.08	2.065
INF03 (didactic videos)	4.99	1.807	4.59	1.843
INF04 (evaluation of content)	3.93	2.006	3.39	1.867
INF05 (information in different formats)	5.09	1.747	5.00	1.838
INF06 (file recovery)	3.36	2.017	3.05	1.874
INF07 (information sources reliability)	4.04	1.869	3.80	1.847

Source: Authors.

**Table 7:** Content creation tools (CCT).

	Knowledge		Usage	
Item	Mean	St. Desv.	Mean	St. Desv.
CCT01 (open educational resources)	4.09	1.796	3.49	1.78
CCT02 (interactive whiteboard)	4.51	1.932	3.71	1.922
CCT03 (voice recordings)	4.41	2.037	3.45	2.09
CCT04 (presentations)	5.60	1.588	5.21	1.67
CCT05 (augmented reality)	2.83	1.851	2.01	1.426
CCT06 (new products)	4.82	1.919	4.10	2.027
CCT07 (QR codes)	4.36	2.225	3.20	2.133
CCT08 (infographics)	4.76	2.017	4.22	2.000
CCT09 (copyright and licences)	4.01	2.045	3.60	1.986
CCT10 (evaluation tests)	4.66	1.887	4.28	1.955
CCT11 (programming)	3.77	2.113	3.49	2.111
CCT12 (rubrics)	4.14	2.148	3.64	2.107
CCT13 (types of licences)	3.83	2.309	3.19	2.223
CCT14 (didactic videos)	4.54	1.907	3.96	1.928
CCT15 (gamification techniques)	3.80	2.050	3.17	1.976
CCT16 (content enrichment)	5.01	1.889	4.76	1.903

Source: Authors.

**Table 8:** Communication tools (COM).

	Knowledge		Usage	
Item	Mean	St. Desv.	Mean	St. Desv.
COM01 (digital technology projects)	4.80	1.942	4.31	2.025
COM02 (software at school)	5.69	1.575	5.50	1.632
COM03 (online communication)	5.80	1.532	5.56	1.624
COM04 (behaviour and etiquette)	4.74	1.921	4.61	1.925
COM05 (social networks)	5.68	1.677	3.22	1.889
COM06 (digital identity management)	3.69	1.961	4.01	1.949
COM07 (experiences and educational research)	4.34	1.820	4.05	2.174
COM08 (collaborative learning)	4.73	2.100	5.15	1.854

Source: Authors.

**Table 9:** Tools associated with safety (SAF).

	Knowledge		Usage	
Item	Mean	St. Desv.	Mean	St. Desv.
SAF01 (device/document protection)	5.02	1.839	4.85	1.906
SAF02 (technological recycling)	4.50	1.986	4.08	2.046
SAF03 (data removal)	4.44	2.104	4.15	2.151
SAF04 (virus threat protection)	4.50	2.067	4.43	2.137
SAF05 (rules on responsible use)	4.81	1.779	4.50	1.84
SAF06 (protection of information)	4.33	2.109	4.17	2.106
SAF07 (basic energy saving)	4.70	2.058	4.43	1.958
SAF08 (control distracting modes)	3.58	1.83	3.23	1.707

Source: Authors.

**Table 10:** Cluster groups as regards the knowledge and use of communication tools (COM).

	K-G1	K-G2	U-G1	UG2	U-G3
Frequency	25.69%	74.31%	35.78%	40.37%	23.85%
Means	3.00	5.56	3.00	5.78	4.78
*AG*	50.64	43.63	48.77	42.27	45.77
*EX*	21.21	12.43	18.79	11.09	14.62
*SX* (M, W)	25%, 75%	45.7%, 54.3%	33.3% 66.7%	40.9%, 59.1%	50%, 50%
*PR* (B, C)	82.1%, 17.9%	74.1%, 25.9%	84.6%, 15.4%	77.3%, 22.7%	61.5%, 38.5%
*TC* (PF, P)	14.3%, 85.7%	12.3%, 87.7%	12.8%, 87.2%	15.9%, 84.1%	7.7%, 92.3%
*LC* (R, U)	25%, 75%	39.5%, 60.5%	35.9%, 64.1%	40.9%, 59.1%	26.9%, 73.1%
*KS* (BFP, ESO)	78.5%, 21.4%	66.7%, 33.3%	76.9%, 23.1%	56.8%, 43.2%	80.8%, 19.2%

Source: Authors.

**Table 11:** Cluster groups as regards the knowledge and use of content creation tools (CCT).

	K-G1	K-G2	U-G1	U-G2
Frequency	36.70%	63.30%	41.28%	58.72%
Means	2.56	5.31	5.06	2.69
*AG*	48.65	43.56	43.43	46.66
*EX*	18.3	12.59	12.48	16.28
*SX* (M, W)	32.5%, 67.5%	44.9%, 55.1%	45.5%, 54.5%	37.5%, 62.5%
*PR* (B, C)	87.5%, 12.5%	69.6%, 30.4%	75%, 25%	76.6%, 23.4%
*TC* (PF, P)	12.5%, 87.5%	13%, 87%	13.6%, 86.4%	12.5%, 87.5%
*LC* (R, U)	32.5%, 67.5%	37.7%, 62.3%	40.9%, 59.1%	32.8%, 67.2%
*KS* (BFP, ESO)	75%, 25%	66.7%, 33.3%	59.1%, 40.9%	76.6%, 23.4%

Source: Authors.

**Table 12:** Cluster groups as regards the knowledge and use of information access and management tools. (INF).

	K-G1	K-G2	K-G3	U-G1	U-G2	U-G3
Frequency	18.35%	38.53%	43.12%	37.62%	33.95%	28.44%
Means	2	6	4	5.43	2.57	4.29
*AG*	50.35	42.86	45.64	43.44	47.51	45.58
*EX*	19.8	11.43	15.43	12.78	16.84	14.65
*SX* (M, W)	20%. 80%	50%, 50%	40.4%, 59.6%	46.3%, 53.7%	27%, 73%	48.4%, 51.6%
*PR* (B, C)	95%, 5%	78.6%, 21.4%	66%, 34%	78%, 22%	73%, 27%	77.4%, 22.6%
*TC* (PF, P)	15%, 85%	14.3%, 85.7%	10.6%, 89.4%	14.6%, 85.4%	16.2%, 83.8%	6.5%, 93.5%
*LC* (R, U)	30%, 70%	35.7%, 64.3%	38.3%, 61.7%	36.6%, 63.4%	32.4%, 67.6%	38.7%, 61.3%
*KS* (BFP, ESO)	85%, 15%	64.3%, 35.7%	67.1%, 31.9%	63.4%, 36.6%	70.2%, 29.7%	77.4%, 22.6%

Source: Authors.

**Table 13:** Cluster groups as regards the knowledge and use of safety tools (SAF).

	K-G1	K-G2	U-G1	U-G2
Frequency	37.62%	62.38%	38.53%	61.47%
Means	2.63	5.63	2.50	5.12
*AG*	48.51	43.57	48.6	43.45
*EX*	18.49	12.4	18.57	12.25
*SX* (M, W)	26.8%, 73.2%	48.5%, 51.5%	28.6%, 71.4%	47.8%, 52.2%
*PR* (B, C)	78%, 22%	75%, 25%	76.2%, 23.8%	76.1%, 23.9%
*TC* (PF, P)	14.6%, 85.4%	11.8%, 88.2%	16.7%, 83.3%	10.4%, 89.6%
*LC* (R, U)	31.7%, 68.3%	38.2%, 61.8%	33.3%, 66.7%	37.3%, 62.7%
*KS* (BFP, ESO)	75.6%, 24.4%	66.1%, 33.8%	73.8%, 26.2%	67.2%, 32.8%

Source: Authors.

**Table 14:** Cluster groups as regards the knowledge and use of problem solving tools (PST).

	K-G1	K-G2	U-G1	U-G2
Frequency	29.36%	70.64%	35.78%	64.22%
Means	2.67	5.67	2.75	5.25
*AG*	50.72	43.23	48.56	43.69
*EX*	21.09	12.02	18.92	12.33
*SX* (M, W)	25%, 75%	46.8%, 53.2%	25.6%, 74.4%	48.6%, 51.4%
*PR* (B, C)	84.4%, 15.6%	72.7%, 27.3%	76.9%, 23.1%	75.7%, 24.3%
*TC* (PF, P)	12.5%, 87.5%	13%, 87%	15.4%, 84.6%	11.4%, 88.6%
*LC* (R, U)	28.1%, 71.9%	39%, 61%	30.8%, 69.2%	38.6%, 61.4%
*KS* (BFP, ESO)	78.2%, 21.9%	66.3%, 33.8%	71.8%, 28.2%	68.6%, 31.4%

Source: Authors.

## Data Availability

Not applicable.

## Appendix A. Questionnaire. Items Groups

	COMMUNICATION TOOLS
COM01	Projects at my school related to digital technologies
COM02	Software available in my school (grades, attendance, communication with family, contents, homework evaluation, etc.).
COM03	Tools for online communication: forums, instant messaging, chats, videoconferencing…
COM04	Basic rules of behaviour and etiquette in online communication in the educational context.
COM05	Social networks or learning communities for sharing information and educational content (e.g., Facebook, Twitter, Google+, or others).
COM06	Forms of digital identity management in the educational context.
COM07	Experiences or educational research of others that can provide me with content, ideas, strategies, for my teaching.
COM08	Tools for shared or collaborative learning (e.g., blogs, wikis, specific platforms such as Edmodo or others).
	CONTENT CREATION TOOLS
CCT01	Open educational resources (OERs).
CCT02	The software of the interactive whiteboard of my school.
CCT03	Tools to create voice recordings (podcast).
CCT04	Tools for creating presentations.
CCT05	Tools for content based on augmented reality.
CCT06	The potential of ICTs to program and create new products (tools, Apps, contents…)
CCT07	Tools to produce QR codes (Quick Response).
CCT08	Tools to facilitate learning such as: infographics, interactive graphics, concept maps, timelines, etc.
CCT09	Sources for locating copyright regulations and licenses of use.
CCT10	Tools to develop evaluation tests.
CCT11	The basic logic of programming, understanding its structure and simple modification of digital devices and their configuration.
CCT12	Tools for developing rubrics.
CCT13	Different types of licenses to publish my content (copyright, copy left and creative commons).
CCT14	Tools for the creation of didactic videos.
CCT15	Tools that help to use gamification techniques in the learning process.
CCT16	Tools for reworking or enriching content in different formats (e.g., texts, tables, audio, images, videos, etc.).
	INFORMATION ACCESS AND MANAGEMENT TOOLS
INF01	Internet browsing strategies (e.g., searching, filtering, use of operators, specific commands, use of search operators, etc.).
INF02	Information management strategies (use of bookmarks, information retrieval, classification, etc.).
INF03	Specific channels for the selection of didactic videos.
INF04	Rules or criteria to critically evaluate the content of a website (updates, citations, sources, etc.).
INF05	Strategies for searching, locating, and selecting information in different media or formats (text, video, etc.).
INF06	Tools for recovering deleted, damaged, inaccessible, or formatted files, etc.
INF07	Criteria for assessing the reliability of information sources, data, digital content, etc.
	PROBLEM SOLVING TOOLS
PST01	Solutions for management and storage in the “cloud”, file sharing, granting access privileges, etc. (e.g., Drive, OneDrive, dropbox, or others).
PST02	Basic solutions to technical problems arising from the use of digital devices in the classroom.
PST03	Tools to help address diversity in the classroom.
PST04	Spaces for training and updating my digital competence.
PST05	Tools to carry out evaluation, tutoring, or monitoring of students.
PST06	Basic computer maintenance tasks to avoid possible malfunctions (e.g., updates, cache, or disk cleaning, etc.).
PST07	Ways to update myself and incorporate new devices, apps, or tools in my work.
PST08	Creative didactic activities to develop digital competence in students.
PST09	Compatibility of peripherals (microphones, headsets, printers, etc.) and their connectivity requirements.
PST10	Options for combining digital and non-digital technology to find solutions in the teaching-learning process.
PST11	Digital resources adapted to the centre’s educational project.
PST12	Peer-to-peer problem solving.
	SAFETY TOOLS
SAF01	Device or document protection system (access control, privileges, passwords, etc.).
SAF02	Recycling points to reduce the impact of technological debris on the environment (unused devices, cell phones, printer toner, batteries, etc.).
SAF03	Ways to remove data/information, when necessary, that you are responsible for about yourself or others.
SAF04	Protection from virus threats, malware, etc., for devices.
SAF05	Rules on the responsible and healthy use of digital technologies.
SAF06	Protection of information (names, images, etc.) relating to people in your immediate environment (classmates, students, etc.).
SAF07	Basic energy saving measures.
SAF08	Ways to control distracting modes of use of technology.

## Appendix B. Spearman Correlation Matrices

**Table A1:** Spearman correlation matrix.

	SX	AG	PR	TC	LC	EX	KS
*SX*							
*AG*	0.016						
*PR*	0.153	−0.125					
*TC*	0.131	0.043	0.022				
*LC*	0.088	0.035	−0.031	−0.229 *			
*EX*	0.053	0.753 **	−0.117	0.100	0.089		
*KS*	−0.076	−0.105	−0.026	0.182	0.034	−0.162	

Source: Authors. *p* value: ** < 0.05; * < 0.10.

**Table A2:** Spearman correlation matrix: knowledge of problem solving tools.

	PST01	PST02	PST03	PST04	PST05	PST06	PST07	PST08	PST09	PST10	PST11	PST12
*SX*	−0.075	−0.180	0.022	−0.210 *	−0.204 *	−0.240 *	−0.162	−0.173	−0.217 *	−0.165	−0.098	−0.085
*AG*	−0.244 *	−0.170	−0.297 **	−0.250 **	−0.239 *	−0.081	−0.272 **	−0.255 **	−0.239 *	−0.232 *	−0.211 *	−0.305 **
*PR*	0.163	0.110	0.139	0.139	0.203 *	0.107	0.069	0.089	0.057	0.124	0.138	0.160
*TC*	0.060	0.054	−0.053	−0.051	−0.069	0.009	−0.049	0.002	0.037	0.025	−0.014	−0.013
*LC*	−0.042	−0.058	0.047	−0.079	−0.035	−0.020	−0.034	−0.198 *	−0.062	−0.156	−0.070	0.067
*EX*	−0.241 *	−0.184	−0.115	−0.256 **	−0.202 *	−0.084	−0.256 **	−0.262 **	−0.193 *	−0.199 *	−0.221 *	−0.313 **
*KS*	0.120	0.099	0.080	0.072	0.106	0.139	0.042	0.059	0.015	0.085	0.094	0.127

Source: Authors. *p* value: ** < 0.05; * < 0.10.

**Table A3:** Spearman correlation matrix: knowledge of information access and management tools.

	INF01	INF02	INF03	INF04	INF05	INF06	INF07
*SX*	−0.067	−0.140	−0.220 *	−0.219 *	−0.256 **	−0.125	−0.174
*AG*	−0.136	−0.232 *	−0.285 **	−0.128	−0.177	−0.229 *	−0.136
*PR*	0.172	0.006	0.128	−0.007	0.131	0.093	0.074
*TC*	0.079	0.099	−0.114	−0.115	−0.075	−0.049	−0.156
*LC*	−0.062	−0.007	−0.123	−0.106	−0.099	0.085	−0.042
*EX*	−0.113	−0.088	−0.201 *	−0.270 **	−0.096	−0.170	−0.250 *
*KS*	0.117	0.145	0.034	0.219 *	0.028	0.213 *	−0.005

Source: Authors. *p*-value: ** < 0.05; * < 0.10.

**Table A4:** (**a**,**b**) Spearman correlation matrix: knowledge of content creation tools.

(**a**)								
	**CTT01**	**CTT02**	**CTT03**	**CTT04**	**CTT05**	**CTT06**	**CTT07**	**CTT08**
*SX*	0.081	0.028	−0.169	−0.165	−0.202 *	−0.108	−0.263 **	−0.043
*AG*	−0.128	−0.165	−0.253 **	−0.269 **	−0.159	−0.210 *	−0.197	−0.281 **
*PR*	0.253 *	0.271 **	0.156	0.057	0.111	0.248 *	0.250 *	0.239 *
*TC*	0.077	0.117	0.109	0.011	0.050	−0.080	−0.015	−0.028
*LC*	−0.097	−0.170	−0.239 *	−0.155	−0.058	−0.036	0.015	0.031
*EX*	−0.162	−0.160	−0.206 *	−0.216 *	−0.160	−0.163	−0.160	−0.296 **
*KS*	0.105	0.096	0.132	0.195 *	0.034	0.077	0.109	0.082
(**b**)								
	**CTT09**	**CTT10**	**CTT11**	**CTT12**	**CTT13**	**CTT14**	**CTT15**	**CTT16**
*SX*	−0.109	−0.069	−0.224 *	−0.002	−0.162	−0.161	0.007	−0.131
*AG*	−0.088	−0.109	−0.096	−0.278 **	−0.205	−0.276 **	−0.218 *	−0.229 *
*PR*	0.057	0.099	0.120	0.194	−0.015	0.062	0.137	0.126
*TC*	−0.066	−0.042	−0.058	−0.084	−0.008	−0.009	−0.024	−0.009
*LC*	−0.024	−0.018	−0.039	0.006	−0.158	−0.204 *	−0.008	−0.175
*EX*	−0.137	−0.110	−0.082	−0.284 **	−0.199	−0.238 *	−0.234 *	−0.168
*KS*	0.165	−0.006	0.196	0.006	−0.033	0.001	0.003	−0.037

Source: Authors. *p* value: ** < 0.05; * < 0.10.

**Table A5:** Spearman correlation matrix: knowledge of communication tools.

	COM01	COM02	COM03	COM04	COM05	COM06	COM07	COM08	COM09
*SX*	−0.083	−0.011	−0.087	0.015	−0.203 *	−0.090	−0.184	−0.098	−0.216 *
*AG*	−0.100	−0.230 *	−0.244 *	−0.175	−0.302 **	−0.286 **	−0.172	−0.194 *	−0.244 *
*PR*	−0.011	0.213 *	0.021	0.183	0.145	0.270 *	0.255 **	0.059	0.135
*TC*	0.841	0.581	0.847	0.417	0.935	0.359	0.303	0.730	0.635
*LC*	−0.002	−0.055	−0.132	−0.046	−0.205 *	−0.052	−0.028	−0.084	−0.074
*EX*	−0.080	−0.161	−0.230 *	−0.162	−0.261 **	−0.126	−0.097	−0.151	−0.218 *
*KS*	0.163	−0.021	−0.006	0.070	0.085	0.067	0.158	0.048	0.073

Source: Authors. *p* value: ** < 0.05; * < 0.10.

**Table A6:** Spearman correlation matrix: knowledge of tools associated with safety.

	SAF01	SAF02	SAF03	SAF04	SAF05	SAF06	SAF07	SAF08
*SX*	−0.168	−0.125	−0.200 *	−0.239 *	−0.087	−0.222 *	−0.181	−0.084
*AG*	−0.174	−0.145	−0.137	−0.109	−0.290 **	−0.220 *	−0.123	−0.260 *
*PR*	−0.117	−0.026	0.145	0.043	0.014 *	0.040	0.198 *	0.125
*TC*	−0.037	−0.027	0.028	0.056	−0.036	−0.090	−0.014	−0.066
*LC*	−0.161	−0.004	−0.161	−0.077	−0.092	−0.184	−0.062	−0.061
*EX*	−0.204 *	−0.178	−0.135	−0.058	−0.265 **	−0.188	−0.135	−0.210 *
*KS*	0.091	0.153	0.102	0.083	0.053	0.041	0.075	0.102

Source: Authors. *p* value: ** < 0.05; * < 0.10.

**Table A7:** Spearman correlation matrix: use of problem solving tools.

	PST01	PST02	PST03	PST04	PST05	PST06	PST07	PST08	PST09	PST10	PST11	PST12
*SX*	−0.027	−0.205 *	0.067	−0.156	−0.213 *	−0.239 *	−0.129	−0.176	−0.248 *	−0.079	−0.104	−0.009
*AG*	−0.294 **	−0.213 *	−0.158	−0.284 **	−0.204 *	−0.051	−0.305 **	−0.203 *	−0.202 *	−0.101	−0.121	−0.322 **
*PR*	0.096	0.095	0.090	0.083	0.186	0.045	0.042	0.097	0.045	0.158	0.126	0.016
*TC*	0.129	0.027	−0.063	−0.034	−0.056	−0.008	−0.094	0.049	0.002	−0.011	−0.071	−0.015
*LC*	0.004	−0.077	0.063	−0.034	−0.042	−0.021	−0.082	−0.208 *	−0.083	−0.105	−0.069	0.062
*EX*	−0.257 **	−0.219 *	−0.028	−0.237 *	−0.139	−0.040	−0.252 **	−0.177	−0.126	−0.031	−0.114	−0.296 *
*KS*	0.020	0.109	−0.016	0.065	0.087	0.084	0.086	−0.005	−0.057	0.029	0.083	0.054

Source: Authors. *p* value: ** < 0.05; * < 0.10.

**Table A8:** Spearman correlation matrix: use of information access and management tools.

	INF01	INF02	INF03	INF04	INF05	INF06	INF07
*SX*	0.005	−0.136	−0.068	−0.090	−0.209 *	−0.084	−0.211 *
*AG*	−0.154	−0.122	−0.067	−0.319 **	−0.069	−0.208 *	−0.146
*PR*	0.043	−0.015	0.023	−0.026	0.079	0.070	−0.013
*TC*	0.073	0.049	−0.029	−0.148	−0.097	−0.090	−0.199
*LC*	−0.089	0.010	−0.099	−0.082	−0.123	0.049	−0.085
*EX*	−0.141	−0.036	−0.007	−0.223 *	−0.014	−0.164	−0.152
*KS*	0.058	0.099	−0.060	0.115	−0.096	0.124	−0.100

Source: Authors. *p* value: ** < 0.05; * < 0.10.

**Table A9:** (**a**,**b**) Spearman correlation matrix.: use of content creation tools.

(**a**)								
	**CTT01**	**CTT02**	**CTT03**	**CTT04**	**CTT05**	**CTT06**	**CTT07**	**CTT08**
*SX*	0.069	0.090	−0.065	−0.015	−0.060	−0.085	−0.162	−0.007
*AG*	−0.050	−0.117	−0.122	−0.325 **	−0.212 *	−0.194	−0.173	−0.296 **
*PR*	0.247 *	0.270 **	0.012	0.037	0.013	0.078	0.000	0.106
*TC*	0.016	0.035	0.119	0.009	−0.056	−0.071	0.014	−0.045
*LC*	0.000	−0.076	−0.193	0.011	−0.011	−0.002	−0.135	0.087
*EX*	−0.054	−0.124	−0.113	−0.237 *	−0.226 *	−0.035	−0.129	−0.234 *
*KS*	0.151	0.003	−0.003	0.180	−0.041	0.010	0.027	0.072
(**b**)								
	**CTT09**	**CTT10**	**CTT11**	**CTT12**	**CTT13**	**CTT14**	**CTT15**	**CTT16**
*SX*	−0.016	−0.052	−0.171	0.020	−0.060	−0.098	0.084	−0.076
*AG*	−0.187	−0.193 *	−0.089	−0.267 **	−0.177	−0.164	−0.238 *	−0.223 *
*PR*	0.082	0.197 *	0.122	0.267 **	0.012	−0.009	0.013	0.072
*TC*	−0.070	0.000	−0.119	−0.135	−0.099	0.034	0.030	0.052
*LC*	−0.070	−0.076	−0.022	0.012	−0.183	−0.150	−0.082	−0.196 *
*EX*	−0.170	−0.109	−0.064	−0.304 **	−0.156	−0.133	−0.246 *	−0.130
*KS*	0.137	−0.026	0.224 *	0.084	−0.088	0.031	0.008	−0.041

Source: Authors. *p* value: ** < 0.05; * < 0.10.

**Table A10:** Spearman correlation matrix: use of communication tools.

	COM01	COM02	COM03	COM04	COM05	COM06	COM07	COM08	COM09
*SX*	−0.059	−0.022	0.012	0.086	−0.038	−0.029	−0.127	0.025	−0.221 *
*AG*	−0.180	−0.212 *	−0.265 **	−0.143	−0.339 **	−0.246 *	−0.188	−0.195	−0.269 **
*PR*	−0.022	0.259 **	0.043	0.200 *	0.034	0.216 *	0.272 **	−0.012	0.124
*TC*	−0.044	0.075	0.043	−0.144	−0.122	−0.183	−0.112	−0.045	−0.046
*LC*	0.021	−0.071	−0.140	−0.058	−0.027	−0.020	−0.004	−0.001	−0.111
*EX*	−0.076	−0.181	−0.189 *	−0.120	−0.225 *	−0.039	−0.121	−0.138	−0.219 *
*KS*	0.120	−0.055	0.019	−0.007	0.132	−0.078	0.139	0.157	−0.002

Source: Authors. *p* value: ** < 0.05; * < 0.10.

**Table A11:** Spearman correlation matrix: use of tools associated with safety.

	SAF01	SAF02	SAF03	SAF04	SAF05	SAF06	SAF07	SAF08
*SX*	−0.112	−0.015	−0.154	−0.186	−0.015	−0.155	−0.138	0.026
*AG*	−0.238 *	−0.140	−0.130	−0.117	−0.322 **	−0.220 *	−0.094	−0.274 **
*PR*	0.120	0.023	−0.077	0.012	0.086	0.101	0.150	0.058
*TC*	0.027	−0.062	−0.005	0.025	−0.042	−0.040	−0.079	−0.143
*LC*	−0.154	−0.032	−0.092	−0.047	−0.100	−0.195	−0.101	−0.010
*EX*	−0.200 *	−0.156	−0.102	−0.036	−0.259 **	−0.154	−0.089	−0.196
*KS*	0.046	0.012	0.106	−0.041	−0.001	0.037	−0.016	0.060

Source: Authors. *p* value: ** < 0.05; * < 0.10.

## Appendix C. ANOVA Analysis. Item Groups

**Table A12:** ANOVA: knowledge and use of problem solving tools.

	Knowledge		Use	
	F	Sig.	F	Sig.
PTS01	97.096	0.000	48.682	0.000
PTS02	210.564	0.000	218.669	0.000
PTS03	46.507	0.000	21.204	0.000
PTS04	108.251	0.000	65.930	0.000
PTS05	221.553	0.000	146.953	0.000
PTS06	97.096	0.000	78.486	0.000
PTS07	147.921	0.000	113.016	0.000
PTS08	84.497	0.000	73.749	0.000
PTS09	168.998	0.000	147.707	0.000
PTS10	184.238	0.000	131.365	0.000
PTS11	92.517	0.000	65.334	0.000
PTS12	71.201	0.000	38.975	0.000

**Table A13:** ANOVA: knowledge and use of information access and management tools.

	Knowledge		Use	
	F	Sig.	F	Sig.
INF01	57.999	0.000	31.291	0.000
INF02	72.283	0.000	105.877	0.000
INF03	70.939	0.000	47.377	0.000
INF04	124.521	0.000	61.635	0.000
INF05	118.445	0.000	81.863	0.000
INF06	52.390	0.000	38.640	0.000
INF07	83.777	0.000	54.344	0.000

**Table A14:** ANOVA: knowledge and use of content creation tools.

	Knowledge		Use	
	F	Sig.	F	Sig.
CCT1	21.363	0.000	11.985	0.001
CCT2	48.748	0.000	15.043	0.000
CCT3	117.474	0.000	99.810	0.000
CCT4	104.470	0.000	37.652	0.000
CCT5	47.164	0.000	30.868	0.000
CCT6	150.648	0.000	109.603	0.000
CCT7	129.873	0.000	87.192	0.000
CCT8	183.080	0.000	69.662	0.000
CCT9	72.719	0.000	68.353	0.000
CCT10	159.399	0.000	76.025	0.000
CCT11	40.547	0.000	22.422	0.000
CCT12	80.794	0.000	47.802	0.000
CCT13	71.961	0.000	69.206	0.000
CCT14	85.265	0.000	45.680	0.000
CCT15	53.892	0.000	34.584	0.000
CCT16	78.793	0.000	61.402	0.000

**Table A15:** ANOVA: knowledge and use of communication tools.

	Knowledge		Use	
	F	Sig.	F	Sig.
COM01	66.822	0.000	34.753	0.000
COM02	107.851	0.000	42.058	0.000
COM03	98.715	0.000	46.417	0.000
COM04	85.915	0.000	55.813	0.000
COM05	142.526	0.000	42.795	0.000
COM06	61.545	0.000	25.989	0.000
COM07	76.639	0.000	52.433	0.000
COM08	143.110	0.000	88.206	0.000
COM09	189.556	0.000	49.947	0.000

**Table A16:** ANOVA: knowledge and use of safety tools.

	Knowledge		Use	
	F	Sig.	F	Sig.
SAF01	86.101	0.000	81.735	0.000
SAF02	58.112	0.000	27.516	0.000
SAF03	266.285	0.000	174.341	0.000
SAF04	111.217	0.000	108.158	0.000
SAF05	122.915	0.000	82.059	0.000
SAF06	105.161	0.000	79.911	0.000
SAF07	55.572	0.000	41.515	0.000
SAF08	96.470	0.000	52.658	0.000
